# Long-term patient-reported outcomes following laparoscopic cholecystectomy: A prospective multicenter observational study

**DOI:** 10.1097/MD.0000000000021683

**Published:** 2020-08-28

**Authors:** In Woong Han, Hyeon Kook Lee, Dae Joon Park, Yoo Shin Choi, Seung Eun Lee, Hongbeom Kim, Wooil Kwon, Jin-Young Jang, Huisong Lee, Jin Seok Heo

**Affiliations:** a Division of Hepatobiliary-Pancreatic Surgery Departments of Surgery, Samsung Medical Center, Sungkyunkwan University School of Medicine; b Department of Surgery, Ewha Womans University College of Medicine; c Department of Surgery, Chung-Ang University College of Medicine; d Department of Surgery, Seoul National University Hospital, Seoul National University College of Medicine, Seoul; e Department of Surgery, Dongguk University College of Medicine, Goyang, South Korea.

**Keywords:** laparoscopic cholecystectomy, patient-reported outcome, quality of life

## Abstract

Several studies have reported short-term results for post-cholecystectomy symptoms and quality of life (QoL). However, reports on long-term results are still limited. This study aimed to identify risk factors affecting short- and long-term patient-reported outcome (PRO) following laparoscopic cholecystectomy.

From 2016 to 2017, a total of 476 patients from 5 institutions were enrolled. PRO was examined using the Numeric Rating Scale (NRS) pain score and the Gastrointestinal (GI) QoL Index questionnaire at postoperative 1 month and 1 year.

Most of patients recovered well at postoperative 1 year compared to postoperative 1 month for the NRS pain score, QoL score, and GI symptoms. A high operative difficulty score (HR 1.740, *P* = .031) and pathology of acute or complicated cholecystitis (HR 1.524, *P* = .048) were identified as independent risk factors for high NRS pain scores at postoperative 1 month. Similarly, female sex (HR 1.571, *P* = .003) at postoperative 1 month and postoperative complications (HR 5.567, *P* = .001) at postoperative 1 year were independent risk factors for a low QoL. Also, age above 50 (HR 1.842, *P* = .001), female sex (HR 1.531, *P* = .006), and preoperative gallbladder drainage (HR 3.086, *P* = .001) were identified as independent risk factors for GI symptoms at postoperative 1 month.

Most patients showed improved long-term PRO measurement in terms of pain, QoL, and GI symptoms. There were no independent risk factors for long-term postoperative pain and GI symptoms. However, postoperative complications were identified to affect QoL adversely at postoperative 1 year. Careful and long-term follow up is thus necessary for patients who experienced postoperative complications.

## 1 Introduction

Since its introduction in 1986, laparoscopic cholecystectomy (LC) has become more widely used and is now considered the treatment of choice for various gallbladder (GB) diseases.^[1–5]^ However, after cholecystectomy, patients often experience various symptoms from the immediate postoperative period to even years after, which can independently predict changes in prognosis, quality of life (QoL), and functional status.^[1,6–11]^ As a result, it is important to recognize that patient-reported outcomes (PRO) incorporate postoperative pain, and various gastrointestinal (GI) symptoms in addition to QoL.^[12–14]^ The Gastrointestinal Quality of Life Index (GIQLI) is 1 of the most widely used questionnaires for the objective measurement of QoL in GI surgery, and its use is validated in gallstone disease.^[13,15–17]^ The European Association for Endoscopic Surgery also recommends the GIQLI questionnaire for the evaluation of QoL for GB disease^[18]^; thus, it should be utilized as a vital measure of outcome for studies on cholecystectomy. There have been various reports that anatomical factors may contribute to PRO and these include sphincter of Oddi dysfunction,^[19,20]^ cystic-duct remnant neuroma,^[21]^ and retained cystic-duct remnant calculi.^[22]^ However, there is limited and inconsistent information available about PRO in these patients.^[23,24]^ For these reasons, the clinical management of these patients is frequently without an evidence-based approach. The purpose of this prospective multicenter observational study is to analyze which factors have the greatest impact on short- and long-term PRO including various postoperative symptoms and QoL using the GIQLI questionnaire following cholecystectomy.

## 2 Methods

### 2.1 Patients

Patients over 18 years with suspected GB diseases were evaluated at the outpatient clinic or emergency room. After a thorough examination that included a physical examination, laboratory testing, and abdominal imaging such as ultrasonography, or a computed tomography scan, the patients who were diagnosed with symptomatic gallstone disease, cholecystitis, GB polyp, or early GB cancer with LC were enrolled. Patients who underwent combined surgery with other gastrointestinal organs were excluded, as were patients with planned radical cholecystectomy for GB cancer, or those with lack of informed consent. All the operations were conducted by experienced biliary surgeons. Operative difficulty was assessed using GB adhesion, distension or contraction, access, severe sepsis or complication, and time to identify the cystic artery or duct.^[25]^ Postoperative complications consisted of bile duct injury (BDI), bleeding, surgical site infection, fluid collection or biloma, bile leak, bile duct obstruction, or bowel injury. LC was performed by single or multiport methods at all institutes.

### 2.2 Study design

**Figure 1:**
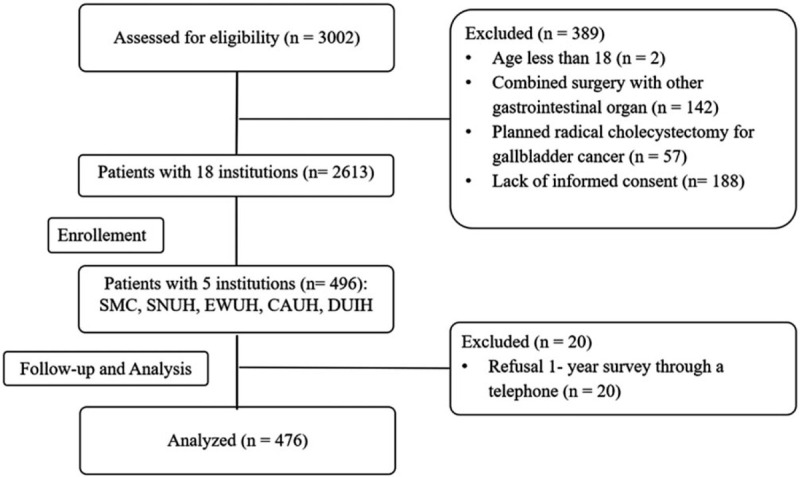
Patients flow according to STROBE statement. CAUH = Chung-Ang University Hospital, DUIH = Dongguk University Ilsan Hospital, EWUH = Ewha Womans University Hospital, SMC = Samsung Medical Center, SNUH = Seoul National University Hospital.

This prospective multicenter observational study evaluated risk factors affecting short- and long-term PRO after LC. Between October 2016 to March 2017, a total of 3,002 patients in 18 institutions were screened for eligibility for the Korea Surgical Improvement Program. Among them, 496 consecutive patients were observed prospectively at 5 tertiary referral centers which were Samsung Medical Center (SMC), Ewha Womans University Hospital, Chung-Ang University Hospital, Seoul National University Hospital, and Dongguk University Ilsan Hospital in South Korea. After exclusion of 20 patients who refused the survey, the results from 476 patients were placed into final analysis (Fig. 1). The Institutional Review Board at each hospital approved the study protocol (*SMC No. 2013–10-122-001*). This study was also registered under clinicaltrials.gov (*NCT02983474*) as a part of Korea Surgical Improvement Program before patient recruitment commenced.

### 2.3 Patient- reported outcome measurements

PROs were evaluated with postoperative pain using the Numeric Rating Scale (NRS) pain score and GI symptoms and QoL using the GIQLI questionnaire at postoperative 1 and 12 months. The survey of PRO at postoperative 1 month was performed at the outpatients’ clinic whereas the survey at postoperative 1 year was performed through a telephone. The NRS pain score is a segmented numeric version of the visual analog scale in which a respondent selects a whole number (0–10 integers) that best reflects the intensity of their pain.^[26,27]^ GIQLI is an instrument that was designed in the early 1990 s by Eypasch, et al^[28]^ to assess health-related QoL in clinical studies of GI disease and in daily clinical practice. The GIQLI questionnaire for GI symptoms consisted of 19 questions. Each question consists of 5 response categories. Questions are scored using a response scale ranging from 0 (worst appraisal) to 4 (best appraisal) points for each question. The questionnaires were self-administered, and patients were given privacy and time to complete the survey. A trained nurse was available for patients that required help in completing the surveys. Outcomes were assessed prospectively by dedicated study nurses who submitted the data to a web-based database (MDB, Seoul, KOR).

### 2.4 Risk factor analysis for patients- reported outcomes

All of the study population was analyzed according to age, sex, body mass index, preoperative percutaneous transhepatic GB drainage (PTGBD), previous abdominal surgery, emergent surgery, operative difficulty, pathology, postoperative complications, and postoperative hospital stay for evaluating short- and long-term pain, QoL, and GI symptoms (Table 1).

**Table 1:**
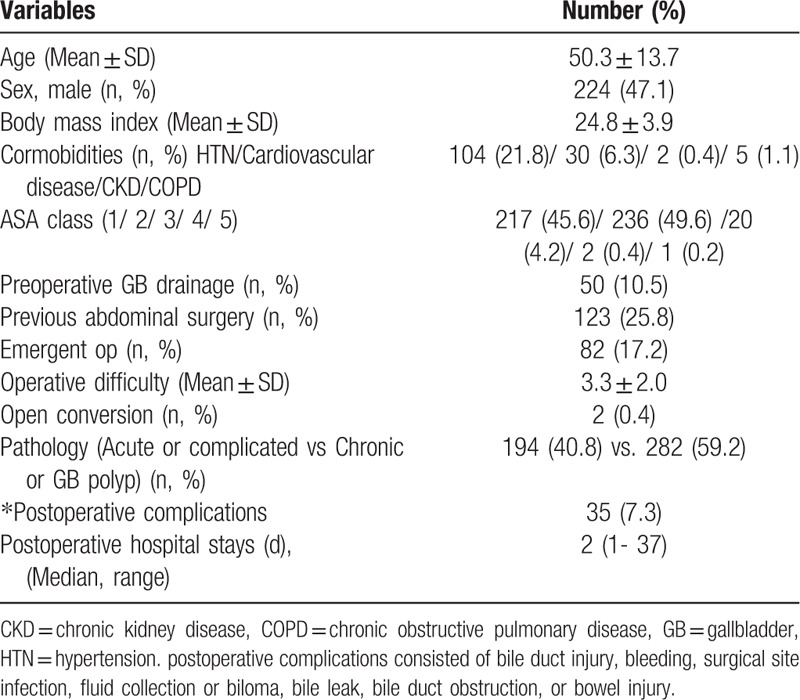
Patients characteristics of enrolled patients.

### 2.5 Statistical analysis

The data were analyzed using SPSS ver. 25.0 (SPSS, Chicago, IL). Continuous and normally distributed variables are presented as the mean ± standard deviation. Continuous parameters in each group were compared using the independent *t*-test, and categorical parameters using the Chi-square test or Fisher exact test. Multivariate analysis was performed using a proportional hazards regression model including a 95% confidence interval (CI) and *P*- value. *P*-values of .05 or less were considered statistically significant.

## 3 Results

### 3.1 Patient characteristics and overall status of PRO measurement

**Figure 2:**
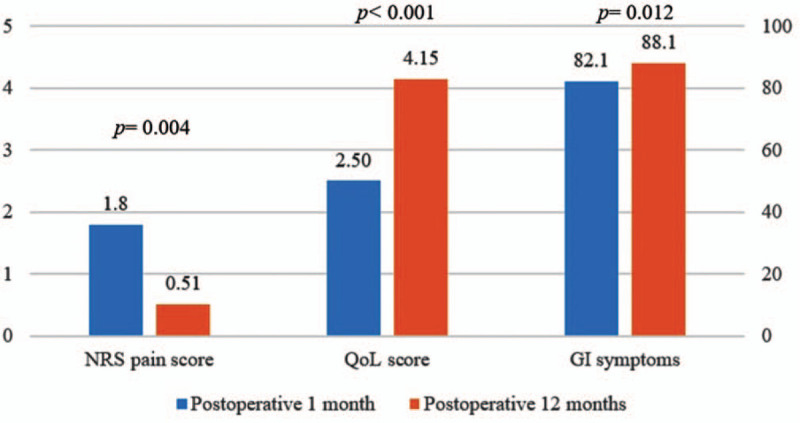
Overall status of patients reported outcomes. GI = gastrointestinal, NRS = Numeric Rating Scale, QoL = quality of life.

The mean age of the study population was 50.3 years, and women- to men ratio was 1.14: 1. The mean operative difficulty score was 3.3 ± 2.0, and median postoperative hospital stays were 2 days (range 1–37 days) (Table 1). Most of the patients reported improved long-term PRO measurement compared to postoperative 1 months in terms of NRS pain score (.51 vs 1.80, *P* = .004), QoL score (4.15 vs. 2.50, *P* < .001), and GI symptoms (88.1 vs 82.1, *P* = .012) (Fig. 2).

### 3.2 NRS pain score

Based on the NRS pain score at postoperative 1 month, women, preoperative PTGBD insertion, emergent operation, high operative difficulty score, pathology of acute or complicated cholecystitis, and a longer hospital stay were identified as short-term risk factors for postoperative pain after univariate analysis (Table 2). Similarly, emergent operation was the only risk factor for long-term follow-up of pain (Table 2). After multivariate analysis, high operative difficulty score (HR 1.740, 95% CI 1.489- 4.119, *P* = .031) and pathology of acute or complicated cholecystitis (HR 1.524, 95% 1.004–2.315, *P* = .048) were identified as independent risk factors for NRS pain score at postoperative 1 month (Table 3). No independent risk factor was identified for long-term follow-up of pain (Table 3).

**Table 2:**
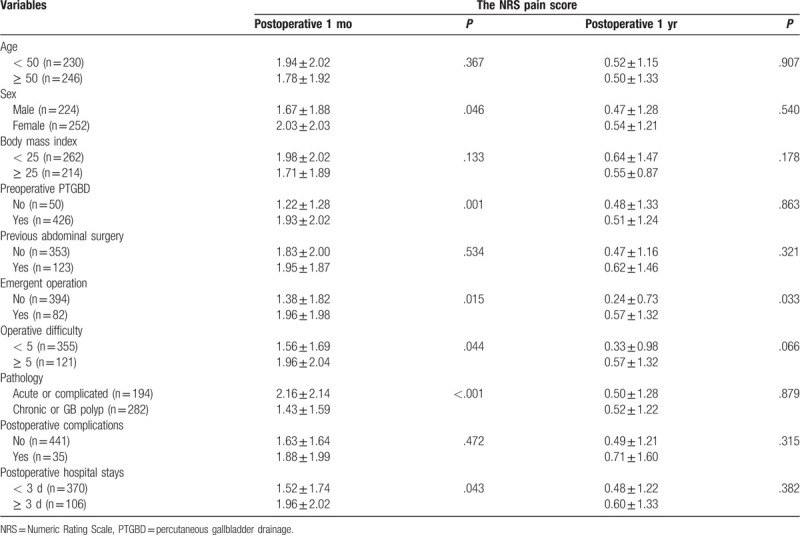
The NRS pain score at postoperative 1 and 12 mo.

**Table 3:**
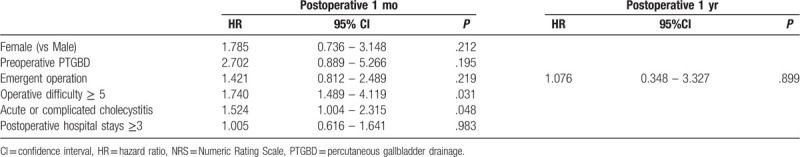
Multivariate risk factor analysis of pain.

### 3.3 QoL score using the GIQLI questionnaire

After univariate analysis, older age and women were identified as risk factors for QoL at postoperative 1month, whereas older age, preoperative PTGBD, emergent operation, high operative difficulty score, and postoperative complications were risk factors at postoperative 12 months (Table 4). Multivariate analysis revealed that female sex (HR 1.571, 95% CI 1.395- 2.826, *P* = .003) at postoperative 1 month and postoperative complications (HR 5.567, 95% CI 2.019- 15.350, *P* = .001) at postoperative 1 year were independent risk factors for lower QoL (Table 5).

**Table 4:**
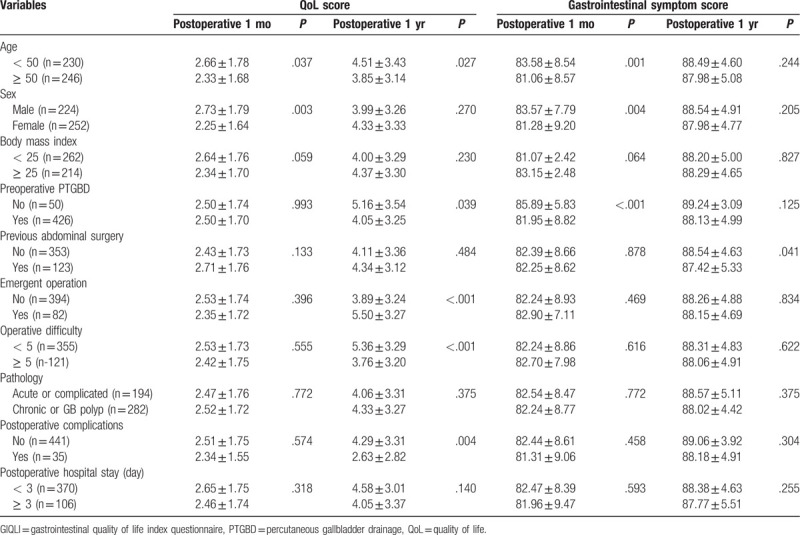
QoL score and gastrointestinal symptoms using GIQLI questionnaire.

**Table 5:**
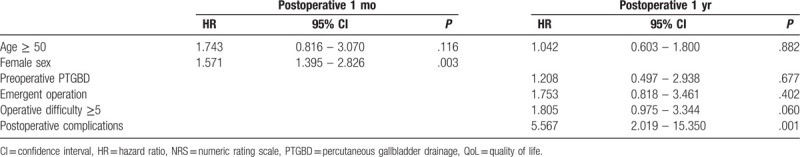
Multivariate risk factor analysis of QoL.

### 3.4 Gastrointestinal symptoms using the GIQLI questionnaire

Similarly, older age, female sex, and preoperative PTGBD were risk factors for GI symptoms at postoperative 1 month, and a history of previous abdominal surgery is a risk factor for long-term follow-up (Table 4). Also, age over 50 (HR 1.842, 95% CI 1.269- 2.673, *P* = .001), female sex (HR 1.531, 95% CI 1.055–2.227, *P* = .006), and preoperative GB drainage (HR 3.086, HR 1.554- 6.129, *P* = .001) were identified as independent risk factors for GI symptoms at postoperative 1 month (Table 6). No independent risk factor was identified for gastrointestinal symptoms at postoperative 1 year (Table 6).

**Table 6:**
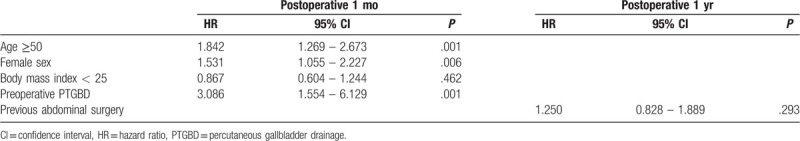
Multivariate risk factor analysis of gastrointestinal symptoms.

## 4 Discussion

When LC is recommended, many patients wonder about the relief of their symptoms including pain and the occurrence of new symptoms after removing the GB.^[29,30]^ As a result, PRO measurement has been useful as a significant determinant of patient satisfaction following cholecystectomy.^[12,13]^ The GIQLI is 1 of the most widely used and validated questionnaires for the objective measurement of QoL including GB disease.^[13,15–17]^ In this prospective multicenter study, most patients showed improved long-term PRO measurement of NRS pain score, QoL score, and GI symptoms using the GIQLI questionnaire compared to short-term PRO measurement (Fig. 2). This positive result following cholecystectomy is consistent with previous published literature.^[11,12,24,31]^ In the case of severe complication such as BDI, several studies reported that extremely long-term follow-up of 8 to 12 years was needed to improve QoL after cholecystectomy.^[14,32,33]^ Also, other studies suggested that the occurrence of a BDI has a great impact on the patient's physical and mental QoL even after excellent functional outcome following repair.^[34,35]^ In this study, postoperative complication including BDI was identified as the only independent risk factor to affect QoL adversely at postoperative 1 year after LC (Tables 4 and 5). As a result, careful and long-term follow up for more than 1 year is necessary for patients who experienced postoperative complications. Cholecystectomy is associated with several physiological changes in the upper GI tract, which may account for persistence of symptoms or worsening QoL after GB removal besides abdominal pain.^[1,7,9,10,30]^ In this study, female sex was an independent risk factor for short-term QoL and GI symptoms after multivariate analysis (Tables 5 and 6). The prevalence of gallstones is known to be higher in female sex,^[17,36]^ and functional causes of abdominal symptoms, such as irritable bowel syndrome,^[37]^ are also more common among women and could possibly resemble gallstone related symptoms. It is therefore not unlikely that for a certain proportion of female patients with GB diseases, cholecystectomy might have had little or no positive effect on QoL or GI symptoms.^[17]^ Postoperative pain is a well-known major determinant for QoL in patients with cholecystectomy, and it is not uncommon with a prevalence of 30% to 50%.^[3,6–11,31]^ After multivariate analysis, high operative difficulty score and pathology of acute or complicated cholecystitis were identified as independent risk factors for the NRS pain score at postoperative 1 month (Tables 2 and 3). We previously reported that a score indicative of higher difficulty in performing LC, in the absence of other definite visceral organ damage, was an independent risk factor in developing short-term postoperative pain.^[29]^ This may be because difficulty in dissection of the triangle formed by the common bile duct, cystic duct, and liver (Calot's triangle) may cause intraoperative nerve damage innervating the visceral structures.^[8,29]^ Preoperative PTGBD has been widely used and has had the benefits of a low complication rate, being a simple operation with early symptom relief and improvements in cases of acute cholecystitis.^[38,39]^ Despite these positive effects on the management of patients, the effects of PTGBD on operative duration and open conversion rates reflecting surgical difficulties have not been identified clearly in patients with acute cholecystitis.^[39]^ Also, as far as we know, the relationship between preoperative PTGBD and PRO has not yet been reported. In this study, preoperative PTGBD was identified as an independent risk factors for GI symptoms at postoperative 1 month (Table 6). As a result, it is worthy to consider short-term symptomatic management in patients who have had preoperative PTGBD. This study has some potential limitations. First, the survey at postoperative 1 year was performed through the telephone which could cause recall bias. Second, in spite of being conducted in a prospective multicenter manner, the study period was relatively short and population number was not large. Thus selection bias cannot be ruled out. A future prospective nationwide study is necessary to evaluate QoL with extremely long-term follow up. In conclusion, most patients reported improved long-term PRO measurement in terms of NRS pain score, QoL score, and GI symptoms using the GIQLI questionnaire. There were no independent risk factors for long-term postoperative pain and gastrointestinal symptoms. However, postoperative complication was identified to affect QoL adversely at postoperative 1 year. Careful and long-term follow up is needed for patients who experienced postoperative complications.

## Author contributions

**Conceptualization:** In Woong Han, Hyeon Kook Lee, Jin-Young Jang, Huisong Lee, Jin Seok Heo.

**Data curation:** In Woong Han, Hyeon Kook Lee, Dae Joon Park, Yoo Shin Choi, Seung Eun Lee, Hongbeom Kim, Wooil Kwon, Jin-Young Jang, Huisong Lee, Jin Seok Heo.

**Formal analysis:** In Woong Han, Hyeon Kook Lee, Dae Joon Park, Yoo Shin Choi, Seung Eun Lee, Hongbeom Kim, Wooil Kwon.

**Funding acquisition:** In Woong Han, Hyeon Kook Lee, Jin Seok Heo.

**Investigation:** In Woong Han, Hyeon Kook Lee, Jin Seok Heo.

**Methodology:** In Woong Han, Hyeon Kook Lee, Dae Joon Park, Yoo Shin Choi, Seung Eun Lee, Hongbeom Kim, Wooil Kwon, Jin-Young Jang, Huisong Lee, Jin Seok Heo.

**Project administration:** In Woong Han, Hyeon Kook Lee, Jin Seok Heo.

**Resources:** In Woong Han, Hyeon Kook Lee, Dae Joon Park, Jin-Young Jang, Jin Seok Heo.

**Software:** In Woong Han, Hyeon Kook Lee, Jin Seok Heo.

**Supervision:** In Woong Han, Hyeon Kook Lee, Dae Joon Park, Yoo Shin Choi, Seung Eun Lee, Hongbeom Kim, Wooil Kwon, Jin Seok Heo.

**Validation:** In Woong Han, Hyeon Kook Lee, Dae Joon Park, Jin-Young Jang, Huisong Lee, Jin Seok Heo.

**Visualization:** In Woong Han, Hyeon Kook Lee, Jin Seok Heo.

**Writing – original draft:** In Woong Han, Hyeon Kook Lee, Jin Seok Heo.

**Writing – review and editing:** In Woong Han, Hyeon Kook Lee, Dae Joon Park, Yoo Shin Choi, Seung Eun Lee, Hongbeom Kim, Wooil Kwon, Jin-Young Jang, Huisong Lee, Jin Seok Heo.
